# Trends in smoking during pregnancy by socioeconomic characteristics in the United States, 2010–2017

**DOI:** 10.1186/s12884-020-2748-y

**Published:** 2020-01-23

**Authors:** Sunday Azagba, Lauren Manzione, Lingpeng Shan, Jessica King

**Affiliations:** 1 Department of Family and Preventive Medicine, Division of Public Health, University of Utah, 375 Chipeta Way, Salt Lake City, UT 84108 USA; 2 Department of Health & Kinesiology, University of Utah, Salt lake city, USA

**Keywords:** Maternal cigarette use, Smoking during pregnancy, Tobacco, Pregnancy, Prevalence

## Abstract

**Background:**

Maternal smoking during pregnancy remains a public health concern in the United States (US). We examined whether the prevalence of smoking during pregnancy decreased between 2010 and 2017 and how trends differed by demographic subgroups.

**Methods:**

We used 2010–2017 data from the National Center for Health Statistics. Rao-Scott Chi-Square tests were performed to compare characteristics between smoking and nonsmoking groups. Cochran–Armitage tests and logistic regression were used to assess overall changes in the prevalence of smoking during pregnancy over time and changes for age, race, and educational attainment subgroups.

**Results:**

The prevalence of smoking during pregnancy decreased from 9.2% in 2010 to 6.9% in 2017. In 2017, the prevalence was highest among women aged 20–24 (9.9%), American Indian/Alaskan Natives (15%), and those with a high school diploma or General Educational Development (GED) (12.2%). The prevalence was lowest among women younger than 15 (1.7%), Asian/Pacific Islanders (1%), and those who had a master’s degree and higher (0.3%). Prevalence did not decrease significantly over time in the 35–39 age group (4.5 to 4.4%; *p* = 0.08), and increased dramatically for women with less than a high school diploma from 10.2 to 11.8%; *p* < 0.0001.

**Conclusions:**

Smoking prevalence during pregnancy in the US is declining, but is highest among younger women (20–24), American Indian/Alaska Natives, and women with a high school diploma or GED. In addition, the prevalence has increased for women with the least education. Targeted research and tobacco control interventions could help address the specific needs of these high-risk subpopulations.

## Introduction

Prenatal cigarette exposure is a leading cause of infant mortality [1]. Despite the well-documented risks of tobacco use, maternal smoking during pregnancy remains prevalent. Of mothers who gave birth in 2016, 7.2% reported being a smoker [2]. Smoking during pregnancy endangers not only the mother’s health (e.g., risk of cancer, cardiovascular disease), but also the health of her unborn child (e.g., risk of premature birth weight, congenital disabilities, sudden infant death syndrome, and metabolic disorders) [2, 3].

Though prenatal smoking remains a significant problem, the prevalence of smoking during pregnancy has been declining for several decades. A national study in the United States (US) found an overall decrease in smoking during pregnancy from 25.7% in 1985, to 10.1% in 2014 [4]. Similar patterns have been observed in Canada, with a prior study showing a steady decline in maternal smoking during pregnancy from 1992 to 2008 [5]. Evidence indicates that certain subpopulations of pregnant women are more likely to smoke during pregnancy. A Canadian study showed that trends in maternal smoking during pregnancy were not consistent across groups with different educational backgrounds [5]. In addition, a 2007–2016 US study found higher smoking prevalence among rural women of reproductive age compared to their urban counterparts [6]. The results of another US study suggest that women who were aged 20–24, American Indian/Alaska Natives, high school educated or less, and had Medicaid coverage had the highest prevalences of smoking before, during, and after pregnancy [7]. These subgroups were also among those with the lowest prevalences of quitting smoking during pregnancy [7].

There is evidence that smoking cessation after becoming pregnant is associated with a reduced risk of preterm birth [8]. In past research, women who were younger than 20, Asian/Pacific Islander, had more than 12 years of education, had private insurance, and lived in urban areas were most likely to quit smoking during pregnancy [6, 7]. However, despite a recent increase in prenatal quitting rates, only half of women quit smoking during pregnancy [4]. This suggests additional efforts are needed to target women at the greatest risk of prenatal smoking.

The existing literature provides some insights into important target populations among women who smoke during pregnancy. However, projections estimate that the maternal smoking rate will be about 6.1% by 2020, which is much higher than the Healthy People 2020 target of 1.4% [4, 9]. This disparity highlights the need for continuous surveillance of smoking during pregnancy to support effective cessation programs and other interventions. This study assessed trends of maternal smoking during pregnancy from 2010 to 2017 in the US. We also examined differences in trends by key sociodemographic characteristics (age, race, and educational attainment subgroups).

## Methods

### Data

The Centers for Disease Control and Prevention’s National Center for Health Statistics (NCHS) collects data for a natality public-use file that contains information on all births registered in the 50 US states and the District of Columbia. The NCHS receives natality data as electronic files, prepared from individual records processed by each registration area, through the Vital Statistics Cooperative Program (VSCP). Information on individual records was derived from the US Standard Certificate of Live Birth, which has served for years as the principal source for attaining uniform birth information. In 2003, the VSCP revised birth certificates to improve data quality and standardization and expedite collection and transmission. The VSCP substantially revised the information collected on maternal tobacco use in 2003 to include the daily number of cigarettes used 3 months before pregnancy and in each trimester of pregnancy. All 50 states and the District of Columbia have adopted the revised birth certificate [10]. The natality public-use data include demographic characteristics of both parents, utilization of medical and public resources/programs, maternal lifestyle, and mother and infant health characteristics. For this study, we used the natality public-use data, restricting records using 2003-revised birth certificates with smoking status during pregnancy starting with 2010. Our study sample ranged from 2,620,675 from 33 states and District of Columbia in 2010, to 3,936,643 from all 50 states and District of Columbia in 2017.

### Measures

Smoking during pregnancy was our outcome of interest. Those who smoked during pregnancy were defined as women who reported smoking cigarettes in any trimester of pregnancy. Women with unknown or an unstated status of cigarette smoking in any trimester of pregnancy were classified as “no valid smoking status” and were excluded from the study. The sociodemographic characteristics examined were maternal age, race, and educational attainment. Maternal age was categorized as “Under 15,” “15–19,” “20–24,” “25–29,” “30–34,” “35–39,” “40–44,” and “45 and over.” Race was categorized as “White,” “Black,” “American Indian or Alaskan Native,” and “Asian or Pacific Islander.” Educational attainment was categorized as “Less than a high school degree,” “High school diploma or General Education Development (GED),” “Some college or associate degree,” “Bachelor’s degree,” and “Master’s degree or higher.”

### Statistical analysis

Sociodemographic characteristics were reported with weighted percentages and classified by the smoking status during pregnancy (smoker, nonsmoker). Rao-Scott Chi-Square tests were used to compare characteristics between the two groups. We generated estimates of the prevalence of smoking during pregnancy for each year of data independently. Additionally, estimates were calculated for the full sample, and for age, race, and educational attainment subgroups separately.

In the trend analyses, Cochran–Armitage tests were used to assess the statistical significance of changes in the prevalence of smoking during pregnancy over time. We also conducted Cochran–Armitage tests to examine the statistical significance of the change in smoking prevalence by subgroup (i.e., age, race, and educational attainment). Logistic regression with the interaction between year and stratification criteria was used to test the difference in trend between stratified subgroups. All stratified analyses based on educational attainment were restricted to women aged 25 and older to minimize the effects of ongoing education. All tests were two-sided, and *p*-values < 0.05 were considered significant. We performed all data analyses using SAS version 9.4 (SAS Institute, Inc., Cary, NC).

## Results

The distribution of characteristics of all participants according to their smoking status during pregnancy is shown in Table 1. The analysis included 29,912,380 live birth cases from 2010 to 2017; 2,261,826 (8.1%) of the mothers reported smoking during pregnancy. More than three-fourths of women were White (76.2%), and 40.7% had a high school diploma, GED, or less. Smoking women were more likely to be White (83.6% vs. 75.6%) and aged between 15 and 29 years (73.4% vs. 56.0%) compared to nonsmokers. Additionally, smokers were more likely to have a high school diploma or GED or less (66.4% vs. 38.4%) compared to nonsmokers.

**Table 1:** The distribution of characteristics of all women according to their smoking status during pregnancy; aggregated years from 2010 to 2017

	Overall n^a^ (%^b^)	Smoker n^a^ (%^b^)	Nonsmoker n^a^ (%^b^)	*P*-value^c^
Total	27,912,380 (100.0)	2,261,826 (8.1)	25,650,554 (91.9)	
Age				< 0.001
Under 15	21,028 (0.1)	532 (0.0)	20,496 (0.1)	
15–19	185,7349 (6.7)	193,188 (8.5)	1,664,161 (6.5)	
20–24	6,118,273 (21.9)	768,677 (34.0)	5,349,596 (20.9)	
25–29	8,037,230 (28.8)	698,979 (30.9)	7,338,251 (28.6)	
30–34	7,450,554 (26.7)	408,433 (18.1)	7,042,121 (27.5)	
35–39	3,583,835 (12.8)	159,177 (7.0)	3,424,658 (13.4)	
40–44	784,035 (2.8)	31,486 (1.4)	752,549 (2.9)	
45 and over	60,076 (0.2)	1354 (0.1)	58,722 (0.2)	
Race				< 0.001
White	21,280,913 (76.2)	1,891,554 (83.6)	19,389,359 (75.6)	
Black	4,372,566 (15.7)	296,856 (13.1)	4,075,710 (15.9)	
American Indian/Alaskan Native	307,120 (1.1)	50,974 (2.3)	256,146 (1.0)	
Asian/Pacific Islander	1,951,781 (7.0)	22,442 (1.0)	1,929,339 (7.5)	
Educational attainment				< 0.001
Less than 12 years	4,342,229 (15.7)	585,466 (26.0)	3,756,763 (14.8)	
High school diploma or GED	6,894,215 (25.0)	907,369 (40.4)	5,986,846 (23.6)	
Some college or associate’s degree	7,988,791 (29.0)	680,190 (30.2)	7,308,601 (28.8)	
Bachelor’s degree	5,320,424 (19.3)	62,209 (2.8)	5,258,215 (20.7)	
Master’s degree or higher	3,043,790 (11.0)	13,377 (0.6)	3,030,413 (12.0)	
Year				< 0.001
2010	2,620,675 (9.4)	241,782 (10.7)	2,378,893 (9.3)	
2011	3,136,124 (11.2)	280,300 (12.4)	2,855,824 (11.1)	
2012	3,317,588 (11.9)	288,811 (12.8)	3,028,777 (11.8)	
2013	3,392,135 (12.2)	287,250 (12.7)	3,104,885 (12.1)	
2014	3,779,767 (13.5)	316,215 (14.0)	3,463,552 (13.5)	
2015	3,883,535 (13.9)	299,705 (13.3)	3,583,830 (14.0)	
2016	3,936,643 (14.1)	282,788 (12.5)	3,653,855 (14.2)	
2017	3,845,913 (13.8)	264,975 (11.7)	3,580,938 (14.0)	

^a^unweighted frequency

^b^% = weighted column percentage

^c^*P*-value corresponding to the Rao-Scott Chi-Square tests

Table 2 presents the trends of smoking prevalence by age groups. The overall prevalence decreased significantly from 9.2% in 2010, to 6.9% in 2017. The prevalence of smoking during pregnancy significantly decreased during the period in all age groups except among women aged 35–39 (*p* = 0.08). Among different age groups, the 2017 prevalence was highest among women aged 20–24 (9.9%), followed by women aged 15–19 (8.2%) and 25–29 (7.9%). By 2017, the prevalence of smoking during pregnancy was lowest among women younger than 15 (1.7%) and those 45 and older (1.7%). The prevalence difference between the highest and lowest age group decreased from 11.6 percentage points in 2010 to 8.2 percentage points in 2017. Logistic regression results show age was a significant predictor of maternal smoking during pregnancy (*p* < 0.0001). Additionally, the interaction between age and year was statistically significant (*p* < 0.0001), indicating that the trend differed by age groups.

**Table 2:** Trend of the prevalence (%) of smoking during pregnancy, by age, 2010–2017

	Overall	Under 15	15–19	20–24	25–29	30–34	35–39	40–44	45 and over
2010	9.23	2.94	12.45	14.45	9.30	5.66	4.52	4.64	2.90
2011	8.94	2.38	11.84	14.19	9.09	5.65	4.45	4.41	2.75
2012	8.71	2.91	11.15	13.81	9.01	5.66	4.38	4.26	2.52
2013	8.47	2.55	10.64	13.33	8.94	5.67	4.37	4.08	2.06
2014	8.37	2.60	10.21	12.97	9.00	5.69	4.56	4.11	2.53
2015	7.72	2.36	9.27	11.73	8.55	5.40	4.47	3.76	2.13
2016	7.18	2.50	8.52	10.71	8.13	5.18	4.42	3.61	1.95
2017	6.89	1.68	8.25	9.88	7.94	5.15	4.39	3.63	1.71
Cochran–Armitage *p*-value	<.0001	0.01	<.0001	<.0001	<.0001	<.0001	0.08	<.0001	<.0001

Note: Logistic regression results show age was a significant predictor of maternal smoking during pregnancy (*p* < 0.0001). Additionally, the interaction between age and year was statistically significant (*p* < 0.0001), indicating that the trend differed by age groups

**Fig. 1:**
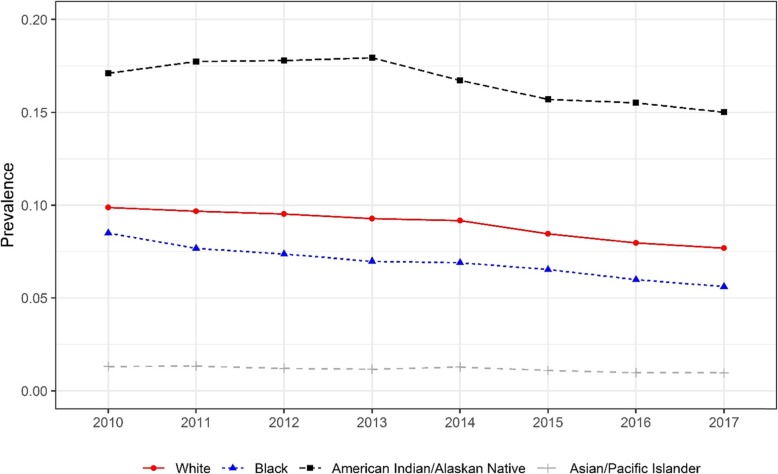
Trend of the prevalence of smoking during pregnancy by race, 2010–2017. Cochran–Armitage tests were used to assess the statistical significance of changes in the trend of the prevalence of smoking during pregnancy stratified by race (*p* < 0.001 for all race subgroups). Logistic regression results show the odds of smoking during pregnancy and whether the trend differed significantly between races (*p* < 0.0001)

The trends of smoking prevalence by race are presented in Fig. 1. The prevalence of smoking during pregnancy decreased significantly between 2010 and 2017 among all races, ranging from less than one percentage point among Asian/Pacific Islander women to three percentage points among Black women. The prevalence of maternal smoking while pregnant was highest among American Indian/Alaskan Native women and lowest among Asian/Pacific Islander women. The gap in prevalence between American Indian/Alaskan Native and Asian/Pacific Islanders remained more than 14 percentage points. Logistic regression results show the odds of smoking during pregnancy as well as the trend differed significantly between races (*p* < 0.0001).

**Fig. 2:**
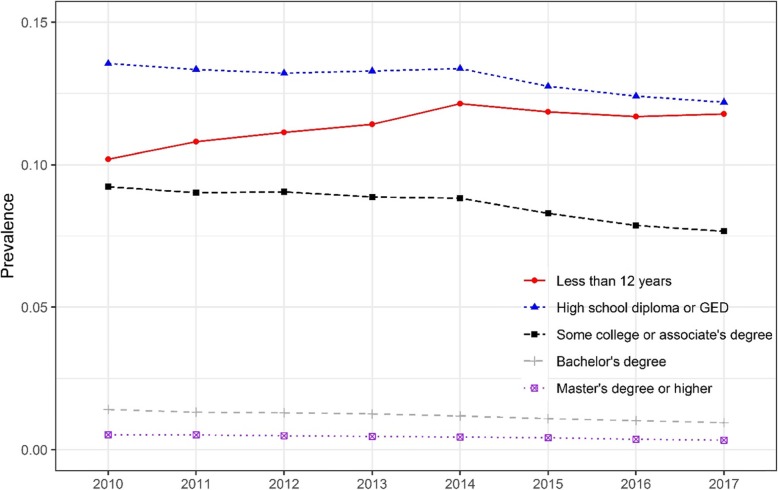
Trend of the prevalence of smoking during pregnancy, by educational attainment, 2010–2017. Cochran–Armitage tests were used to assess the statistical significance of changes in the trend of the prevalence of smoking during pregnancy stratified by educational attainment (*p* < 0.001 for all education subgroups). Logistic regression results show the odds of smoking during pregnancy and whether the trend differed significantly between educational attainment groups (*p* < 0.0001)

Figure 2 presents the trend of smoking prevalence by educational attainment. Smoking prevalence was lowest among women who had a master’s degree or higher, followed by women with a bachelor’s degree. The highest prenatal smoking prevalence was among women with a high school diploma or GED. The prevalence in all educational attainment groups decreased significantly except among women with less than a high school diploma. Over the study period, the prevalence of smoking during pregnancy among women with less than a high school diploma increased significantly from 10.2% in 2010 to 11.8% in 2017. A significantly different trend was found between education attainment groups (*p* < 0.0001). The prevalence difference between women with a high school diploma or GED and women with less than a high school diploma decreased from 3.3 percentage points in 2010 to less than half a percentage point in 2017.

## Discussion

We examined trends in maternal smoking during pregnancy from 2010 to 2017 in the US and examined differences by age, race, and educational attainment. Overall, smoking prevalence steadily decreased from 2010 to 2017. This extends the work of Tong and colleagues who assessed trends in smoking during pregnancy between 2000 and 2010 in the US [7]. Similar findings have been reported elsewhere. For example, Mohsin and colleagues examined trends in smoking during pregnancy between 1994 and 2007 in Australia [11] and found decreases in overall smoking prevalence. The decrease in maternal smoking rates reflects those found in the general population, among both men and women [12]. US adult smoking rates reached an all-time low of 13.7% in 2018, which still surpassed the Healthy People 2020 Goal of 12% [13, 14]. The current study also found important differences in the prevalence of smoking during pregnancy by subgroups.

Our analyses indicate education is a significant risk factor for smoking during pregnancy. The prevalence of smoking while pregnant was lowest among women who had a master’s degree or higher. In contrast, the prevalence was consistently highest among women with a high school diploma or GED. Similarly, a previous study reported that women with less than a high school degree have the highest prevalence of smoking during pregnancy, followed by those with a high school diploma or GED [7]. Prior findings also indicate that women with the lowest levels of education are most likely to smoke before pregnancy and continue smoking during pregnancy [15–17]. In our study, the prevalence increased significantly among women with less than a high school degree, despite decreasing in all other groups. In Canada, the pregnancy smoking prevalence disparity also grew between the groups that were the least and most educated [5]. Future qualitative research may provide insight into reasons for the trend disparity among educational subgroups.

The prevalence of smoking during pregnancy varied by age group in the present study, which steadily decreased between 2010 and 2017 in almost every age group, with the notable exception of those who were 35–39. Smoking prevalence during pregnancy was consistently highest among the 20–24 age group, followed by those aged 15–19. These findings are in line with previous evidence that women younger than 24 had the highest prevalence of smoking while pregnant [7, 11, 15]. We also found that smoking prevalences were lowest in the under 15 and over 45 age groups. Grouping the under 15 women separately from the women aged 15–19 allowed us to observe the significant differences in smoking prevalence between the two groups. Previous studies have also reported lower prenatal smoking prevalence in age groups older than 30 [11, 15], but specifically, grouping women older than 45 allowed us to observe a continued decline in smoking prevalence as age increases past 30.

The current study found that smoking during pregnancy decreased for all race groups, but prevalence varied greatly. The prevalence of smoking during pregnancy was highest among American Indian/Alaskan Native women and lowest among women who are Asian/Pacific Islander, which is similar to prior findings [7]. The general US population reflects this trend, where smoking prevalence remains highest among the American Indian/Alaska Native population and lowest among the Asian population [18]. This disparity in smoking during pregnancy suggests the need for more research and interventions to address the gap in smoking prevalence between race groups. One study found that most American Indian smokers wanted to quit or tried to quit smoking during the previous year, but few were successful [19]. A high unsuccessful quit rate suggests that culturally-specific adaptations of nationally successful smoking cessation strategies are needed to help reach American Indian/Alaska Native populations more effectively, including pregnant women. Though the prevalence is consistently low, the grouping of Asians and Pacific Islanders together because of sample sizes may mask areas of concern among the subgroups. In Hawaii, for instance, Native Hawaiians have the highest prevalence of smoking [20]. Thus, despite pregnancy-specific smoking rates among the Native Hawaiian subgroup, smoking during pregnancy might be a concern among this population. Additionally, this illustrates a need for additional data among Asian and Pacific Islander populations in order to allow for subgroup comparisons.

Our study has several limitations. One limitation is that women who did not reach fetal viability were excluded since the VSCP does not record miscarriages as births. Another constraint is the absence of data about the use of tobacco products other than cigarettes, which is a rising national concern [18]. Additionally, the dataset did not include unique identification for women who may have had multiple births from 2010 to 2017. Despite these limitations, this study provides a clear view of smoking trends during pregnancy and identifies the demographic groups that are most at risk.

## Conclusion

This study assessed trends as well as sociodemographic differences in maternal smoking during pregnancy from 2010 to 2017. We found that the prevalence of smoking during pregnancy is on the decline overall, but is highest among women aged 15–29, American Indian/Alaska Native women, and women with a high school diploma, GED, or less. Furthermore, education is a significant factor, and our data show that smoking prevalence has increased for women with less than a high school diploma. Targeted research, as well as smoking prevention and cessation programs, could help address the specific needs of these high-risk subpopulations.

## Data Availability

The dataset used in this study is publicly available from: https://www.cdc.gov/nchs/nvss/index.htm

## Abbreviations

GED: General Educational Development
NCHS: National Center for Health Statistics
US: United States
VSCP: Vital Statistics Cooperative Program
